# Identification of Novel Leishmania donovani Nucleoside Hydrolase Inhibitors from Banisteriopsis laevifolia Using Affinity Selection-Mass Spectrometry

**DOI:** 10.1021/acsomega.5c01613

**Published:** 2025-05-23

**Authors:** Pamella C. O. de Oliveira, Pedro R. C. Medeiros, Bruno C. B. Marques, Jorge L. S. Simão, Martin Albino, Vanessa G. P. Severino, Claudio Sangregorio, Luzineide W. Tinoco, Marcela C. de Moraes

**Affiliations:** † BioCrom, Organic Chemistry Department, Chemistry Institute, Fluminense Federal University, 24020-141 Niterói, Rio de Janeiro, Brazil; ‡ Laboratory for Analysis and Development of Enzyme Inhibitors, Natural Products Research Institute, Federal University of Rio de Janeiro, 21941-902 Rio de Janeiro, Rio de Janeiro, Brazil; § Institute of Chemistry, Federal University of Goiás, Campus Samambaia, Esperança Avenue, 74690-900 Goiânia, Brazil; ∥ ICCOM-CNR, 50019 Sesto Fiorentino (FI), Italy; ⊥ Department of Chemistry ‘Ugo Schiff’, University of Florence and INSTM, 50019 Sesto Fiorentino (FI), Italy

## Abstract

The identification of enzyme inhibitors from natural sources offers a promising pathway for drug discovery. In this study, affinity selection-mass spectrometry (AS-MS) was employed to screen for inhibitors of Leishmania donovani nucleoside hydrolase (*Ld*NH) from crude extracts of Banisteriopsis laevifolia. The enzyme was immobilized onto magnetic nanoparticles, enabling selective ligand retention and downstream analysis. The leaf extract exhibited significant inhibitory activity, with an IC_50_ value of 0.73 ± 0.09 μg/mL, prompting further exploration. Analytical-scale fractionation and biochromatogram analysis revealed inhibitory regions, while AS-MS facilitated the annotation of nine flavonoid-based ligands, including procyanidins, glycosylated flavonoids, and rutin. The structures of four ligands (isoquercetin, astragalin, rutin, and orientin) were confirmed using commercial standards. Among these, isoquercetin and astragalin demonstrated potent *Ld*NH inhibition with IC_50_ values of 40.2 ± 16.6 and 41.6 ± 8.9 μmol/L, respectively. These findings highlight B. laevifolia as a promising source of bioactive compounds and demonstrate the utility of AS-MS for efficiently identifying enzyme inhibitors in natural libraries.

## Graphical abstract

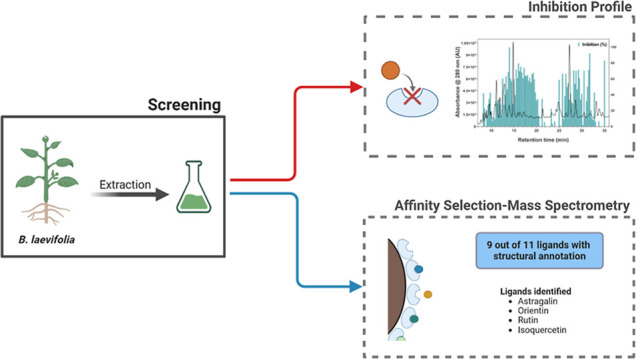

## 1 Introduction

Leishmaniasis is a group of neglected tropical diseases caused by protozoan parasites of the genus Leishmania, transmitted through the bite of infected phlebotomine sandflies. Among its forms, visceral leishmaniasis (VL), also known as kala-azar, is the most severe. Present in over 80 countries and strongly associated with poverty, VL is primarily caused by Leishmania donovani in Asia and Eastern Africa.^1^ Treatment options are limited by high costs, lengthy therapies, drug resistance, and systemic toxicity, underscoring the urgent need for novel therapeutic strategies targeting the Leishmania parasites.^2^

The enzyme nucleoside hydrolase (NH), a key component of the purine salvage pathway in L. donovani, has emerged as a promising target for VL treatment. This enzyme is essential for the parasite’s nucleotide metabolism, catalyzing the hydrolysis of *N*-glycosidic bonds in nucleosides, which are crucial for parasite survival and replication. Notably, NH is absent in mammals, making it an attractive target for developing selective therapeutic agents.^3^

Natural products have proven to be a rich source of bioactive compounds, offering exceptional structural diversity and favorable safety profiles. Their extensive chemical space has significantly contributed to drug discovery, with nearly two-thirds of all FDA approved small drugs since 1981 being derived from or related to natural products.^4^ However, less than 10% of global biodiversity has been explored for biological activity.^5,6^ Recognizing this, natural products have been investigated as antileishmanial agents.

Focusing on L. donovani nucleoside hydrolase (*Ld*NH), various studies have reported the identification of inhibitors from natural product extracts. For example, Nirma et al. identified flavonoids from Leandra amplexicaulis and Urvillea rufescens, with IC_50_ values ranging from 1.1 μmol/L to 197 μmol/L, demonstrating uncompetitive inhibition.^7^ Similarly, Casanova et al. isolated two proanthocyanidins from Ormosia arborea fractions using ^1^H NMR-guided analysis, which exhibited noncompetitive inhibition with IC_50_ values of 25.6 and 28.2 μmol/L.^8^ Tabrez et al. investigated Cassia fistula extracts, combining molecular docking and *in vitro* assays to confirm inhibitory activity against *Ld*NH.^9^ While promising, these studies primarily relied on traditional workflows involving fractionation and purification of bioactive compounds, which are labor-intensive, time-consuming, and often fail to identify minor constituents with potent activity.

To overcome these limitations, affinity selection-mass spectrometry (AS-MS) has emerged as an efficient alternative. This technique takes advantage of the specificity of protein–ligand interactions. In AS-MS, the target protein is incubated with a complex mixture, such as libraries of synthetic or natural compounds. Ligands within the mixture form protein–ligand complexes, while nonligands remain in solution. The protein–ligand complex is separated from the remaining mixture components, and then the ligand is identified following the dissociation of the complex.^10^

AS-MS is particularly promising for screening bioactive compounds in crude natural product extracts. It eliminates the need for exhaustive fractionation and enables the chemical structure annotation of the isolated ligand by searching online MS databases for known natural products or through comparison with reference standards. For novel compounds, sufficient quantities can be isolated for complete structural characterization using techniques such as two-dimensional NMR and MS.^10,11^

In previous work by our research group, immobilized *Ld*NH was used to screen ligands from Moringa oleifera extracts. The enzyme was attached to magnetic particles, facilitating the separation of enzyme-ligand complexes from the nonbinding components present in the extract. Ligand identification was performed indirectly by comparing the extract composition before and after enzyme incubation. Compounds inferred as ligands were identified based on their absence in the chromatogram of the extract following incubation with the enzyme, an approach known as “missing peak.” This approach was necessary because the experimental conditions did not allow for dissociation of the enzyme–ligand complex. Using this approach, de Faria et al. identified seven promising compounds, providing a foundation for the application of AS-MS in the identification of *Ld*NH inhibitors from natural product extracts.^12^

In the present study, we build upon these efforts to develop efficient methods for screening *Ld*NH inhibitors in crude extracts from natural products. Specifically, we present the first study to employ the complete AS-MS workflow—including effective ligand isolation—for the identification of inhibitors targeting *Ld*NH. This approach was applied to Banisteriopsis laevifolia, an underexplored species in the Malpighiaceae family. While several species within this genus exhibit pharmacological activities, such as antioxidant, antibacterial, and monoamine oxidase inhibition, B. laevifolia remains relatively understudied. To date, only a few studies have investigated its antimicrobial and antioxidant properties, leaving its full biological potential largely unexplored. Preliminary analyses suggest its high content of phenolic compounds, flavonoids, and tannins, indicating its potential as a source of novel bioactive molecules. By applying AS-MS, we aimed to identify potential *Ld*NH inhibitors directly from B. laevifolia flower and leaf extracts, further advancing our group’s pursuit of innovative solutions for leishmaniasis treatment.

## 2 Results and Discussion

Natural products are renowned for their structural diversity and pivotal role in drug discovery, offering promising pharmacokinetic properties (ADME).^13^ However, working with natural libraries, such as plant extracts, presents significant challenges due to the labor-intensive and time-consuming nature of metabolite isolation.

AS-MS provides a solution to these challenges by exploiting protein–ligand interactions to isolate ligands from complex natural product libraries efficiently. Ligands with target affinity are selectively retained, enabling their separation from nonbinding components. Immobilizing the target protein on magnetic particles further streamlines the process, facilitating the isolation of protein–ligand complexes while minimizing ligand loss due to complex dissociation.

### 2.1 MNP Synthesis and Characterization

Powder X-ray diffraction (XRD) was used to investigate the crystalline phase of the MNPs. The obtained diffractograms (Figure S1) confirm the presence of the magnetite/maghemite phase. While the diffractograms closely matched the magnetite standard, the presence of maghemite (γ-Fe_2_O_3_), cannot be ultimately excluded based on XRD analysis alone. The absence of significant peaks corresponding to other iron oxide phases further reinforces the presence of a predominantly magnetite/maghemite phase in the synthesized MNPs. Given their magnetic properties, the desired crystalline phase was magnetite (Fe_3_O_4_), for maintaining the desired magnetic behavior, since maghemite presents less preeminent magnetic response.^14^

Transmission electron microscopy (TEM) analysis of MNP evidenced the shape, dispersion, and size distribution of the material. Based on the micrograph (Figure S2), the MNPs presented a mean size of 25 nm. All data regarding MNPs characterization are available in the Supporting Information.

Fe_3_O_4_ magnetic particles offer several advantages as support for protein immobilization, including facile surface functionalization and the ability to efficiently retain the target protein.^15^ Nanometric supports, such as the one used in this work, exhibited some features, such as a high surface-to-volume ratio. For protein immobilization, this means a greater number of anchoring spots on the surface compared with microparticles. Moreover, nanoparticle suspension tends to be more stable than microscale materials, which can positively affect enzymatic function.^16,17^ The functionalization of MNPs using 6-aminohexyl phosphonic acid represents an effective alternative to the conventional silica coating, which is typically more labor-intensive and time-consuming due to its multiple modification steps. This approach ensures strong and stable anchoring sites for enzyme immobilization while simplifying the functionalization process.

### 2.2 Validation of the Optimized LC-DAD Method for Ino/Hypo

To improve the efficiency of inosine (Ino) and hypoxanthine (Hypo) chromatographic separation, a shorter analytical column was evaluated. The original 15 cm column used by de Faria et al. yielded a 12 min runtime.^12^ By switching it with a 5 cm analytical column containing a stationary phase compatible with the previous method, a similar chromatographic resolution was achieved while reducing the runtime to only 3 min (Figure S3). The new method was validated by assessing selectivity, linearity, precision, accuracy, and the limits of detection (LOD) and quantification (LOQ). Comprehensive validation data can be found in the Supporting Information.

Selectivity was assessed by injecting only phosphate-buffered saline (PBS; 20 mmol/L, pH 7.4, 300 mmol/L NaCl), used as the enzymatic reaction blank, which showed no interference in the analyte signal (Figure S4).

The method demonstrated linearity across Hypo concentrations ranging from 1 to 160 μmol/L, represented by the equation *y* = 16018.1*x* + 2665.0 (Figure S5) with a correlation coefficient (*R* ^2^) of 0.9997.

The method was proven to be accurate and precise, with accuracy values ranging from 89.6 and 103.7% and coefficient of variation values (CV) between 1.06 and 11.20%, well within the 15% acceptance threshold (Table S1). The LOD and LOQ were determined to be 0.025 μmol/L and 0.1 μmol/L, respectively.

### 2.3 Immobilization of *Ld*NH onto MNP

Enzymes are highly efficient biocatalysts but are often susceptible to denaturation under varying conditions such as temperature, pH, and the presence of organic solvents.^18^ However, this limitation can be mitigated by immobilizing the enzyme on a magnetic support. This approach not only increases the enzyme stability against changes in temperature, pH, and the presence of organic solvents but also provides greater control over the system, allowing for easy removal from the reaction medium by applying an external magnetic field.^19,20^

In this study, glutaraldehyde acts as an activating and spacer agent, introducing reactive aldehyde groups onto the aminated surface of the synthesized MNPs to enable *Ld*NH immobilization. The enzyme is subsequently immobilized by forming Schiff bases, wherein nucleophilic groups on its surface (such as amine and sulfhydryl groups) react with the aldehyde groups on the activated support. This strategy ensures a covalent and stable attachment of the enzyme to the MNPs. The use of glutaraldehyde is well-established in biochemical applications due to its efficiency, versatility, and compatibility with aqueous systems, making it an ideal choice for this immobilization method.^21^

The immobilization yield was determined by measuring the difference in protein concentration between the initial *Ld*NH solution and the supernatant after immobilization using the Lowry method. The process achieved a high immobilization yield of 89.1 ± 5.88% (*n* = 3), corresponding to approximately 178 μg of *Ld*NH immobilized per mg of synthetic MNP.

### 2.4 Stability Assays

The stability of the *Ld*NH-MNP was evaluated over a 5-month period with samples analyzed in triplicate. The enzyme-coated MNPs were stored at 4 °C when not in use. Figure 1 illustrates the variation in enzymatic activity during this time frame, expressed as a percentage relative to the initial catalytic activity measured immediately after immobilization. To facilitate the interpretation, the activity values were standardized based on the Hypo production at the initial time point, resulting in percentage values, in some cases, above 100%.

**Figure 1:**
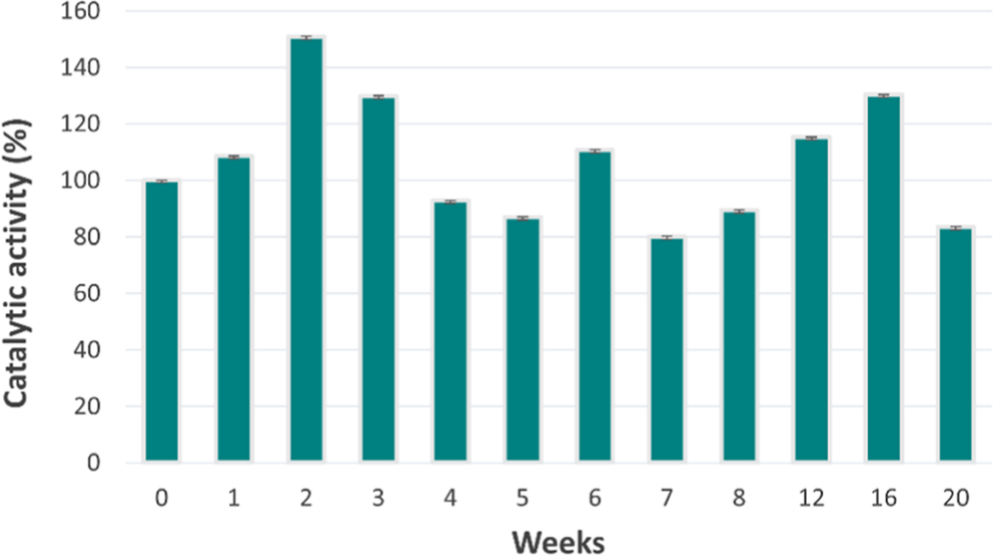
Stability of *Ld*NH-MNPs over time. Enzymatic activity was monitored weekly by weekly quantification of Hypo production.

The *Ld*NH-MNPs system demonstrated high stability, retaining its catalytic activity over the 5-month evaluation period. This result confirms the efficacy of the immobilization approach in preserving the *Ld*NH catalytic activity, enabling its long-term use with minimal activity loss.

### 2.5 Kinetic Assays

The kinetic characterization of *Ld*NH-MNP was performed to determine the apparent Michaelis–Menten constant (*K*_Mapp_), which indicates the enzyme’s affinity for its substrate, inosine (Ino). This parameter is crucial for optimizing inhibition assays, as it determines the substrate concentration at which competitive inhibitors can be most effectively evaluated. Inhibition assays benefit from substrate concentrations close to the *K*_Mapp_, as excessively high concentrations could displace competitive inhibitors from the enzyme active site.

The *Ld*NH-MNP exhibited a *K*_Mapp_ of 486.5 ± 30.0 μmol/L. This value is consistent with previous studies using free *Ld*NH in solution (*K*_Mapp_ = 434 ± 109 μmol/L^22^) and *Ld*NH immobilized onto commercial magnetic microparticles (*K*_Mapp_ = 464.0 ± 53.4 μmol/L^12^). These results indicate that *Ld*NH immobilization onto the synthetic MNP did not affect the enzyme affinity for inosine, preserving its native kinetic behavior. The fitted curve is presented in the Supporting Information (Figure S6).

### 2.6 Functional Assays—Inhibition Studies

Enzymes play a central role in various biological processes, making their regulation essential for maintaining physiological balance. Enzymatic inhibitors, which interact with enzymes to modulate their activity, are invaluable in drug discovery. Nearly half of all commercially available drugs function as enzyme inhibitors.^23^ Small molecules (under 3000 Da) are especially interesting as enzyme inhibitors due to their enhanced absorption and cellular permeability.

Natural extracts are a valuable source of bioactive small molecules. The Banisteriopsis genus has garnered attention due to its wide range of pharmacological properties. For instance, Oliveira et al. reported antifungal activity in Banisteriopsis argyrophylla leaf extracts and fractions, which are rich in flavonoids, including quercetin-3-*O*-α-l-rhamnose, kaempferol-3-*O*-α-l-rhamnose, and their galloyl derivatives. These compounds showed minimum inhibitory concentrations (MICs) ranging from 5.86 to 46.87 μg/mL against Candida albicans, Candida glabrata, and Candida tropicalis.^24^ Additionally, Banisteriopsis caapi has been associated with monoamine oxidase A and B inhibition, as well as antioxidant properties.^25,26^ Wang et al. and Samoylenko et al. identified bioactive constituents such as harmol, harmine, harmaline, and proanthocyanidins, including epicatechin and procyanidin B2, in its extracts.^25,26^

Despite extensive research on certain species within this genus, B. laevifolia remains underexplored. In this context, our study provides valuable insights into the chemical profile and biological activities of B. laevifolia, contributing to the understanding of this underexplored species, and shedding light on its pharmacological potential and chemical diversity.

#### 2.6.1 Evaluation of *Ld*NH Inhibitory Potential of B. laevifolia Leaf and Flower Extracts

Screening assays for B. laevifolia leaf and flower extracts (Bl-EEL and Bl-EEF, respectively) provided inhibition percentages of 99.3 ± 0.11% and 99.2 ± 0.17%, respectively, at a concentration of 200 μmol/L. These results indicate that both extracts are promising sources of *Ld*NH inhibitors.

Dose–response curves were generated for Bl-EEL and Bl-EEF extracts using concentrations ranging from 0.5 to 200 μg/mL (Figure 2). The calculated IC_50_ values were 0.73 ± 0.09 μg/mL for Bl-EEL and 1.5 ± 0.01 μg/mL for Bl-EEF, demonstrating that Bl-EEL exhibited the highest inhibitory potential against *Ld*NH. Consequently, Bl-EEL was selected for further investigation to identify bioactive compounds responsible for the observed activity.

**Figure 2:**
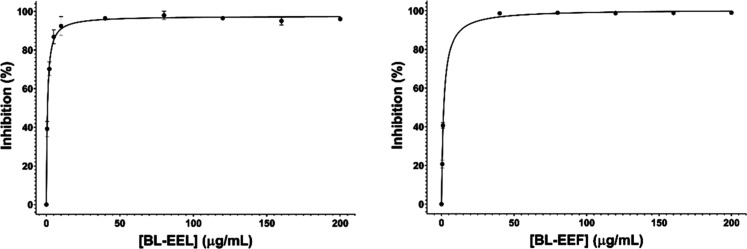
Dose–response curves used to determine the IC_50_ values of B. laevifolia extracts, Bl-EEL (left) and Bl-EEF (right).

#### 2.6.2 *Ld*NH Inhibition Profiling for B. laevifolia Leaf Extract

To identify the bioactive compounds responsible for *Ld*NH inhibition, the chromatographic profile of the B. laevifolia leaf extract (Bl-EEL), which demonstrated the highest inhibitory activity, was analyzed using the method described in Section 4.7.2. The chromatographic profiling using the HPLC-DAD system revealed 102 peaks at 280 nm and 58 peaks at 365 nm.

Fractionation was performed within the 8–35 min range, with fractions collected at 15-s intervals, providing a resolution of 4 data points per minute. The inhibitory profile was visualized as a biochromatogram, plotting the percentage of inhibition for each fraction as a function of retention time (Figure 3). The biochromatogram analysis revealed distinct regions with high inhibitory activity as follows: between 12 and 20 min (41–99%), between 25 and 32 min (39–92%), and at 35–35.5 min (83%), highlighting these regions as associated with the presence of potent *Ld*NH inhibitors.

**Figure 3:**
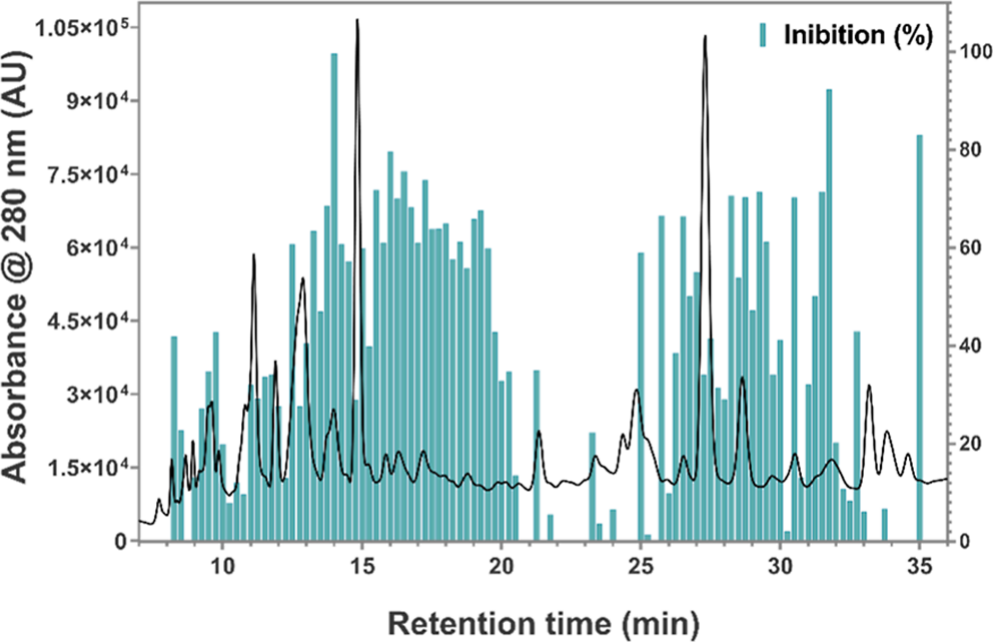
Biochromatogram obtained after fractionation the retention time of 8–35 min monitored at 280 nm wavelength. Black: chromatographic profile of B. laevifolia leaf extract (Bl-EEL) at 10.0 mg/mL; blue: inhibition percentage of each fraction.

The biochromatogram provides valuable insights into the retention times corresponding to the elution of *Ld*NH inhibitors during the chromatographic analysis of the crude extract. However, this method does not enable the direct identification of these compounds, as the retention times with the highest inhibition percentages may result from the coelution of multiple compounds. Therefore, an AS-MS assay was conducted to isolate and identify *Ld*NH ligands present in the Bl-EEL extract.

### 2.7 Identification of *Ld*NH Ligands in B. laevifolia Leaf Extract Using AS-MS

The AS-MS technique is based on the specificity of protein–ligand interaction to isolate ligands present in libraries,^27^ such as the Bl-EEL extract. In this study, the prepared *Ld*NH-MNPs were incubated with Bl-EEL extract, followed by a washing and elution step, as described in Section 4.8.

Figure 4 shows the overlay of the total ion chromatogram (TIC) of Bl-EEL, S_3 active *Ld*NH_ and S_3 control_, in positive mode. The AS-MS assay exhibited selective retention of 11 *Ld*NH ligands, with affinity ratios ≥1.2 (Table S2), eluting between 12.4 and 33.5 min, corresponding to the inhibition regions observed in the biochromatogram (Figure 3).

**Figure 4:**
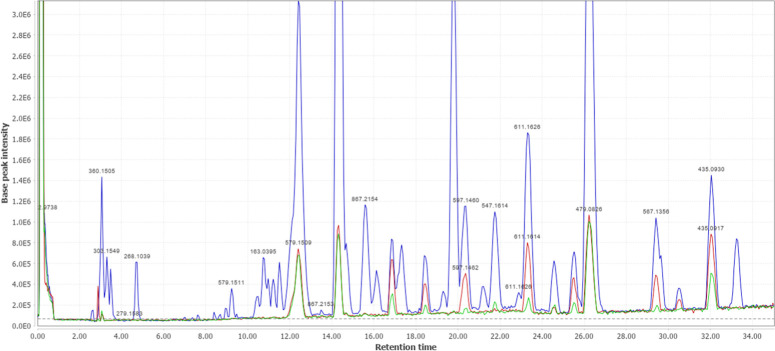
Total ion chromatogram (TIC) of B. laevifolia leaf extract (blue); and the supernatants from ligand elution in the AS-MS assay using active (red) and inactive *Ld*NH (green), up to a retention time of 35 min.

The *Ld*NH ligands were annotated using high-resolution mass spectrometry coupled to liquid chromatography (LC-HRMS). The raw data were processed and analyzed using MZmine software (version 3.9).^28^ The chemical features were compared against virtual spectral libraries available on the Global Natural Products Social (GNPS) platform (http://gnps.ucsd.edu).^29^ This comparison utilized library matches and spectral similarities to facilitate compound annotation, with a cosine threshold ≥ 0.90 and at least 4 shared peaks. Of the 11 ligands analyzed, nine were successfully annotated based on their exact mass and fragmentation patterns, as summarized in Table S2.

The majority of the annotated ligands are flavonoids, including procyanidins and glycosylated flavonoids, which are known for their antioxidant and anti-inflammatory properties and are widely distributed in plants.^30,31^ Procyanidins, in particular, have been extensively studied for their potential health benefits, such as anticancer and antidiabetic effects.^26,32,33^

The ligand annotated as procyanidin at a retention time of 12.4 min showed an intense signal for *m*/*z* 289, characteristic of the catechin monomeric unit, a common core for this class. The annotation of epicatechin for *m*/*z* 291 (RT = 14.3 min) further supports the annotation of procyanidin at *m*/*z* 579, as catechin and epicatechin are well-known monomeric units of procyanidins.^33^

The ligands orientin and isoorientin were assigned to *m*/*z* 449 observed at retention times of 16.8 and 18.4 min, respectively. The fragmentation patterns were consistent with the characteristic fragments resulting from the successive loss of water molecules. For orientin, the fragment at *m*/*z* 431 represents the loss of a single water molecule from *m*/*z* 449, while *m*/*z* 413 corresponds to the loss of two water molecules. Additional fragmentation included *m*/*z* 329, attributed to the loss of C_4_H_6_O_3_ from *m*/*z* 431, and *m*/*z* 299, resulting from the loss of C_5_H_6_O_3_ from *m*/*z* 413, as represented in Figure 5.^34,35^

**Figure 5:**
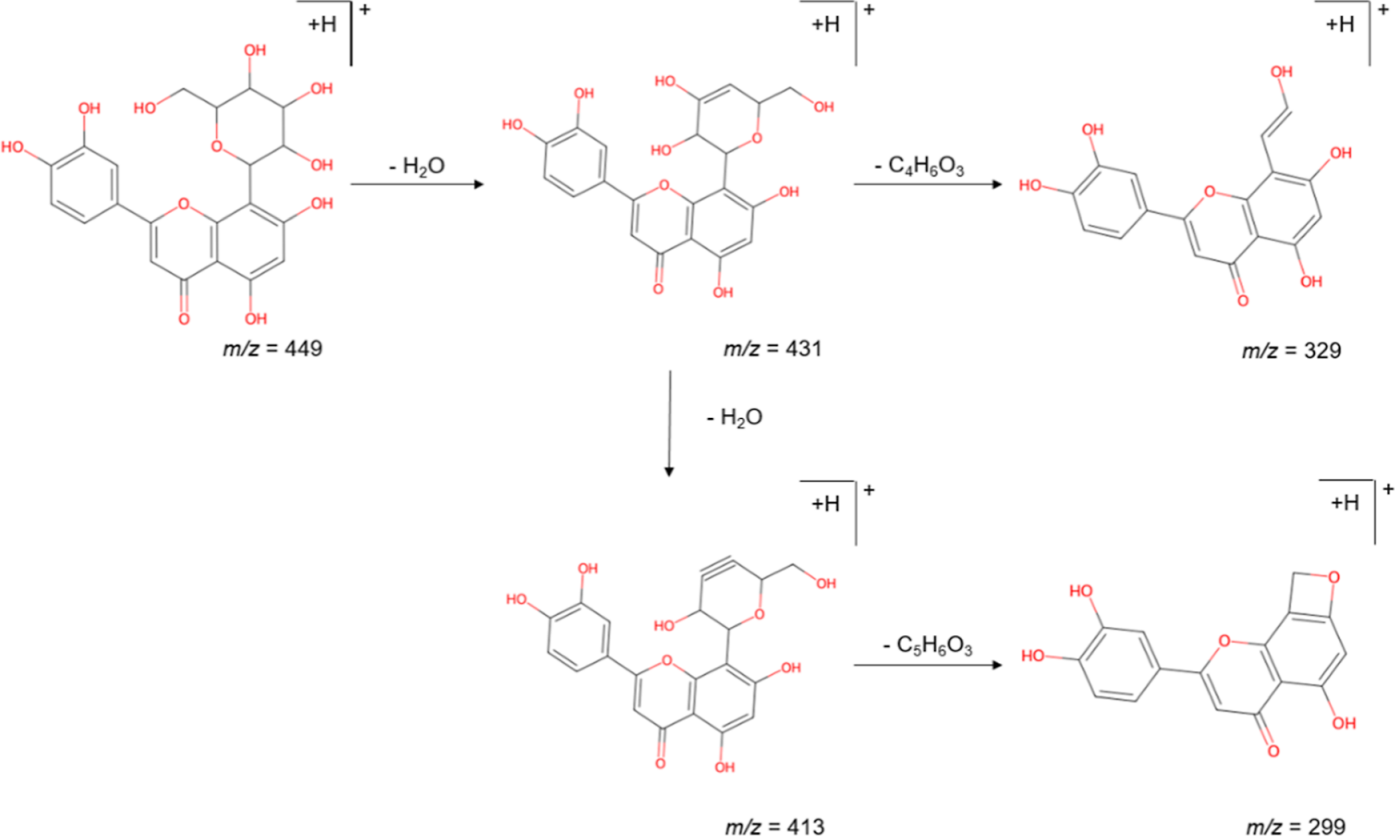
Proposed fragmentation of isomers orientin/isoorientin. Adapted from Wei et al.^34^ This figure is distributed under the terms of the Creative Commons Attribution License (CC BY 4.0).

Another isomer with *m*/*z* 449, at a retention time of 33.5 min, was annotated as astragalin. Its main fragmentation pattern involved the loss of C_6_H_10_O_5_, corresponding to the sugar moiety in the molecular ion *m*/*z* 449, resulting in the fragment *m*/*z* 287.^36^

The compound annotated at a retention time of 23.3 min was identified as rutin, a glycosylated flavonoid belonging to the flavonol class. The presence of fragment *m*/*z* 303, associated with the basic structure of flavonoids, was observed and it was attributed to the loss of two sugar molecules (rhamnose). The fragment *m*/*z* 465 corresponds to the structure after the loss of rhamnose molecule, while the rhamnose oxonium ion, resulting from this loss, was observed in the fragment *m*/*z* 129.^37^

The ligand at 25.5 min was annotated as isoquercetin. The fragmentation of its molecular ion, *m*/*z* 465, undergoes a mass loss of C_6_H_10_O_5_ (162 Da) and generates the fragment *m*/*z* 303, which represents the basic structure of quercetin.^38^

The ligand with a retention time of 26.2 min was tentatively identified as quercetin 3-*O*-glucuronide. This compound exhibited a molecular ion at *m*/*z* 479 and a characteristic fragment ion at *m*/*z* 303, corresponding to the loss of a glucuronic acid moiety (176 Da).^39^

At retention time of 29.39 min, the virtual libraries suggest that the ligand with molecular ion of *m*/*z* 567.1344 has a quercetin moiety—observed in the fragment *m*/*z* 303, associated with two sugar molecules—furanose and ribose rings. The fragment *m*/*z* 133 represents a loss of water from the ribose structure, producing the ion C_5_H_9_O_4_. The following fragments *m*/*z* 115 (C_5_H_9_O_4_–H_2_O) and *m*/*z* 97 (C_5_H_9_O_4_–2H_2_O) represent a consecutive loss of water molecules.^40^

Of the nine annotated ligands, four were commercially obtained—orientin, rutin, isoquercetin, and astragalin—for structural confirmation and inhibition studies. Individual solutions of these ligands were analyzed under the same HPLC-DAD conditions, and their retention times were consistent with those observed in the Bl-EEL chromatogram. This alignment verified the accuracy of the structural annotations. A comparison of retention times is presented in Figure S7, demonstrating the reliability of the identification process.

As shown in Table 1, these ligands exhibited affinity ratio (AR) values ranging from 2.2 to 4.7, indicating moderate to strong interaction with the *Ld*NH enzyme. These AR values suggest selective binding to the enzyme, with minimal nonspecific interactions involving the support material or the inactivated enzyme, as observed in control assays.

**Table 1:** Inhibition Assay of *Ld*NH Ligands Identified through AS-MS Analysis

ligand	inhibition at 100 μmol/L (%)	inhibition at 100 μmol/L (with Triton X-100) (%)	AR
orientin	37.9	30.4	2.3
rutin	58.5	61.2	3.5
astragalin	74.5	66.7	2.2
isoquercetin	80.9	76.0	4.7

To further evaluate their potential as *Ld*NH inhibitors, the four ligands—orientin, rutin, isoquercetin, and astragalin— were evaluated at an initial concentration of 100 μmol/L. The inhibition results are summarized in Table 1. Since flavonoids are notorious assay interference compounds resulting from their promiscuous binding interactions, additional inhibition assays were conducted in the presence of 0.1% Triton X-100 to assess the specificity of their inhibitory effects. The % inhibition values obtained in the presence of the detergent were very similar to those observed in its absence, suggesting that the activity is not due to nonspecific interactions.

Orientin and rutin exhibited moderate inhibition (<65%) at 100 μmol/L, while astragalin and isoquercetin showed stronger inhibitory effects, proceeding to IC_50_ determination. These subsequent assays were performed in the presence of 0.1% Triton X-100. Isoquercetin, a glycosylated form of quercetin, demonstrated potent inhibitory activity, with an IC_50_ value of 40.2 ± 16.6 μmol/L. Astragalin, another glycosylated flavonoid derived from quercetin, also exhibited significant inhibitory activity, with an IC_50_ value of 41.6 ± 8.9 μmol/L. The dose–response curves of astragalin and isoquercetin are presented in Figure 6.

**Figure 6:**
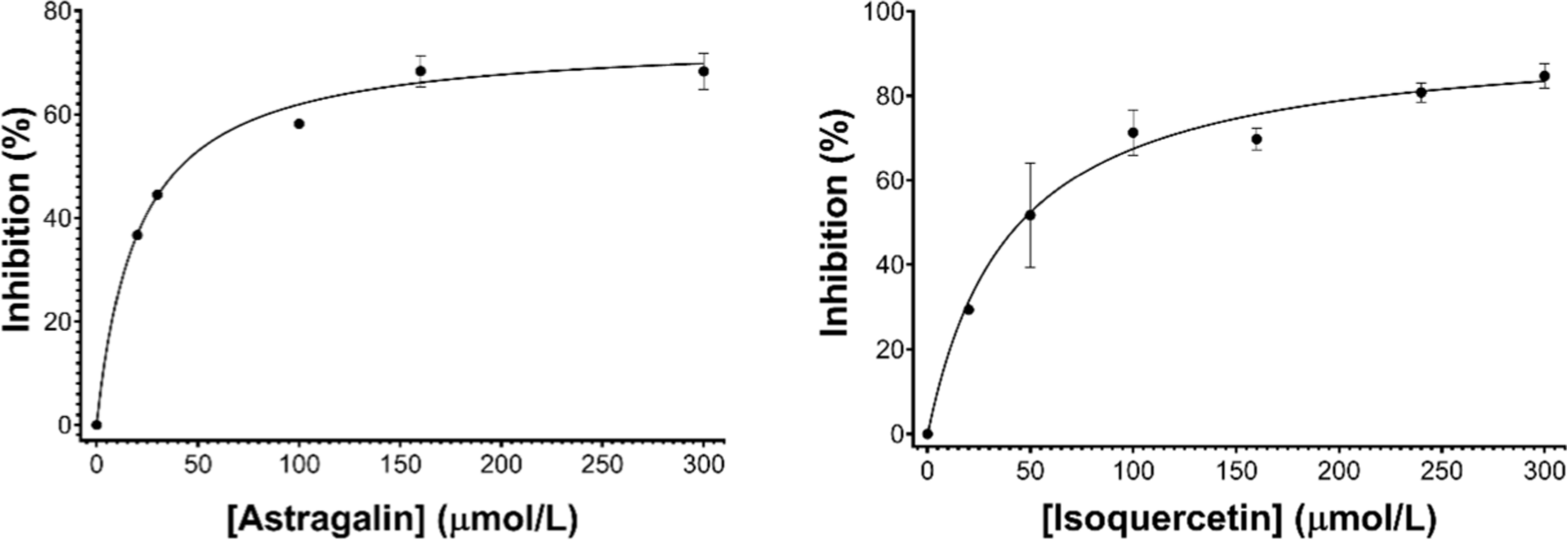
Dose–response curves used to determine the IC_50_ value of astragalin (left) and isoquercetin (right).

These results are consistent with those reported for other natural products previously investigated as *Ld*NH inhibitors. For instance, kaempferol derivatives and proanthocyanidins have shown IC_50_ values as low as 1 μmol/L.^8^ Other *Ld*NH inhibitors identified from natural products, such as tricetin 4-*O*-methyl-flavone, kaempferol 3-*O*-β-d-xylopyranosyl-(1→2)-α-l-rhamnopyranoside, kaempferol 3-*O*-α-l-rhamnoside, and two type-A proanthocyanidins presented IC_50_ values of 1.1 μmol/L, 74.7 μmol/L, 197.4 μmol/L, 25.6 μmol/L, and 28.2 μmol/L, respectively.^7,8^ These compounds, including the ones identified in this study, share structural similarities, particularly the presence of sugar molecules and hydroxyl groups, which are crucial for interaction with metal ions within the enzyme active site.^22^

Building on the promising activity observed for the identified inhibitors, further investigations using *in vitro* studies are necessary to assess their effectiveness against L. donovani protozoa in their amastigote and promastigote forms. Moreover, a detailed exploration of their structure–activity relationships (SAR) could support the design and identification of derivatives with greater potency and optimized pharmacological properties. Additionally, molecular docking studies could provide valuable insights into the binding modes and key molecular interactions between the flavonoids and the active site of *Ld*NH. Such computational approaches would help to rationalize the observed inhibitory activities and guide the development of more selective and potent inhibitors.

Besides the biological potential of the ligands individually, the B. laevifolia itself represents a species that is relatively understudied, and its chemical composition remains largely unknown. This study aims to contribute to our understanding of B. laevifolia chemical profile. Recently, Da Silva et al. evaluated the chemical profile of a hydroethanolic B. laevifolia leaf extract, among others leaves extracts.^41^ By following similar analytical approaches, we aim to identify as many compounds within this plant as possible. Table S3 summarizes all the annotated structures using GNPS present in the Bl-EEL extract used in this work.

Epicatechin, chlorogenic acid, isoquercetrin, quercetin-3-*O*-glucuronide, procyanidins type A and B, and rutin are examples of molecules associated with Banisteriopsis species, including B. laevifolia.^24,26,41,42^ Suggesting that the use of GNPS platform was coherent with previous studies and reliable in the proposed structures.

## 3 Conclusions

This study successfully demonstrated the potential of B. laevifolia as a promising source of *Ld*NH inhibitors. Using AS-MS assays, several compounds—primarily flavonoids such as procyanidins, isoquercetin, astragalin, and rutin—were isolated as *Ld*NH ligands and annotated. Enzyme inhibition assays with orientin, rutin, isoquercetin, and astragalin confirmed their inhibitory activity, validating AS-MS as an effective tool for screening of *Ld*NH inhibitors. Isoquercetin and astragalin exhibited inhibitory potency in the micromolar range, with IC_50_ values of 40.2 ± 16.6 and 41.6 ± 8.9 μmol/L, respectively.

These findings highlight B. laevifolia as a valuable source of bioactive compounds and provide important insights into its chemical composition. Additionally, an easy and effective coating and functionalization step was used for the magnetic nanoparticles in this study, ensuring their suitability for affinity-based screening. Further investigations are warranted to assess the efficacy of these inhibitors against Leishmania parasites. Moreover, molecular docking and SAR studies are planned as future approaches to refine these inhibitors and guide the design of more potent derivatives. The results underscore the critical importance of exploring natural products as a source of new drugs candidates for the treatment of visceral leishmaniasis.

## 4 Materials and Methods

### 4.1 Reagents and Chemicals

The water used to prepare solutions, and the mobile phase was obtained using a MILLI-Q DIRECT 8 system from Millipore Merck (Darmstadt, Germany). Sodium chloride, potassium dihydrogen phosphate, ammonium acetate, dimethyl sulfoxide, glycine, glutaraldehyde (25% in H_2_O), hypoxanthine (Hypo), inosine (Ino), pyridine (≥99%), triethylamine (Et_3_N), oleic acid (90%), dibenzyl ether (99%), oleylamine (≥98%), chloroform, 6-aminohexyl phosphonic acid (AEPA), triton X-100 and ethanol were purchased from Sigma-Aldrich (St. Louis, USA). The metal precursor iron­(III) acetylacetonate (Fe­(acac)_3_, 99%) was obtained from Strem Chemicals (Newburyport, USA). Acetonitrile and methanol both HPLC grade were purchased from J.T.Baker (Xalostoc, Mexico). Orientin and astragalin were purchased from ChemScene (Monmouth Junction, USA) while rutin and isoquercetin were purchased from Sigma-Aldrich (St. Louis, USA).

### 4.2 Plant Material and Extraction

Access to the genetic heritage was registered at the National System for the Management of Genetic Heritage and Associated Traditional Knowledge (SisGen) under code A11AE20. B. laevifolia leaves and flowers were collected in the city of Goiânia, Goiás, Brazil (coordinates: 16°43′25″ S, 49°15′50″ W). The plant was identified by Dr. Aristônio Magalhães Teles, and a voucher specimen (UFG-60052) was deposited in the herbarium of the Federal University of Goiás (UFG), Brazil.

The fresh flowers (618 g) were extracted by maceration with ethanol (3 × 1 L for 3 days each) at room temperature. The resulting extract was filtered and concentrated under reduced pressure using a rotary evaporator, yielding 49.7 g (8.0%) of the ethanolic flower extract (Bl-EEF).

The air-dried leaves (719 g) were pulverized using a knife mill and extracted by maceration with ethanol (3 × 2 L for 3 days each) at room temperature. After extraction, the mixture was filtered, and the solvent was removed under reduced pressure using a rotary evaporator, yielding 79.2 g (11.0%) of the ethanolic extract of the leaves (Bl-EEL).

### 4.3 Magnetic Nanoparticles (MNPs) Synthesis and Characterization

The synthesis of Fe_3_O_4_ magnetic nanoparticles (MNPs) was previously described in the literature.^43,44^ The synthetic route used 8 mmol of Fe­(acac)_3_, 24 mmol of oleic acid, and 24 mmol of oleylamine, dissolved in 60 mL of dibenzyl ether. The reaction mixture was kept under vigorous magnetic stirring and constant N_2_ flow. The reaction medium was heated from 20 to 300 °C over 20 min and held at this temperature for 1 h and 30 min, then rapidly cooled back to room temperature. The resulting suspension was rinsed with ethanol, and the solid product was separated from the dispersion using a permanent magnet.

Before proceeding to enzyme immobilization, the MNPs were amino-functionalized following methods previously described in the literature.^44,45^ For MNP surface modification, 6-aminohexyl phosphonic acid was used to introduce amino groups. The coating exchange was performed using a mass ratio of 1:1 for the MNP and the coating agent. MNP was suspended in chloroform, and 6-aminohexyl phosphonic acid was dissolved in DMSO. In the sequence, they were mixed and kept in an ultrasonic bath for 1 h. Then, the suspension was kept under gentle agitation for 24 h. Lastly, the amino-functionalized MNPs were washed with ethanol and stored in a mixture of ethanol and water (8:2, v/v).

The support characterization evaluated the crystalline phase and size of the synthesized MNPs by powder X-ray diffraction (XRD) analysis using a Bruker D8 Advance (Billerica, USA) and transmission electron microscopy (TEM) analysis using a PHILIPS CM 12 (Eindhoven, The Netherlands).

### 4.4 Optimization of the Chromatographic Method

The activity of the immobilized *Ld*NH was monitored using a chromatographic method that furnished the separation of inosine (Ino) and hypoxanthine (Hypo), the substrate and product of the enzymatic catalysis, respectively. The conditions were based on a method previously developed by our research group, optimized to reduce analysis time.^12^

The optimized Ino/Hypo chromatographic separation used an Ascentis Express C18 column (50 × 4.6 mm, 5 μm), mobile phase containing 1% Et_3_N v/v, acidified with acetic acid, pH (6.0) and MeOH at the proportion of 95:5 in isocratic elution mode at a flow rate of 0.8 mL/min. The total analysis time was 3 min. Analytes were monitored at 249 nm, with an injection volume of 20 μL.

#### 4.4.1 Optimized Chromatographic Method Validation

The new method was validated considering the following parameters: selectivity, linearity, precision and accuracy, limit of quantification (LOQ), and limit of detection (LOD). Selectivity was evaluated through the analysis of a blank sample containing only phosphate-buffered saline (PBS; 20 mmol/L, pH 7.4, 300 mmol/L NaCl). Linearity was verified through an analytical curve of Hypo at a concentration range of 1–160 μmol/L. For this, a 2 mmol/L Hypo stock solution was prepared in PBS, and from this stock solution, concentrations of 1, 2.5, 5, 10, 40, 80, and 160 μmol/L were prepared in PBS in triplicate. The analytical curve was obtained by plotting solution concentration versus peak areas.

Concentrations of 1.2, 50, and 150 μmol/L were used as low, medium, and high concentration controls to study the intra- and interday precision and accuracy of the analytical method. The controls were prepared in quintuplicate and diluted with PBS, using a 2 mmol/L Hypo stock solution. Precision was established by the coefficient of variation (CV) of the replicates, while accuracy was determined as the ratio between the average concentration value obtained from the analytical curve and the reference concentration value. Only CV and accuracy values below 15% were considered acceptable.

The LOQ and LOD were determined using a 10 μmol/L Hypo stock solution to prepare a series of dilutions in PBS. Dilutions at the following concentrations of Hypo were produced: 0.010, 0.025, 0.050, and 0.100 μmol/L, prepared in triplicate. The LOQ was determined as the lowest concentration of analyte at which precision and accuracy values within 15% were obtained. The LOD was determined as the lowest analyte concentration at which the signal-to-noise ratio (S/N) was equal to or greater than 3:1.

### 4.5 *Ld*NH Immobilization onto MNPs

The *Ld*NH enzyme was expressed in Escherichia coli BL21­(DE3) and purified using nickel affinity chromatography. After dialysis, the enzyme was concentrated and stored in phosphate-buffered saline (PBS; 20 mmol/L, pH 7.4, 300 mmol/L NaCl) at −80 °C until use.^22^

The procedure for the covalent immobilization of *Ld*NH on magnetic nanoparticles (MNP) was adapted from the conditions previously described by our group. Briefly, the MNP surface was activated with glutaraldehyde as a cross-linking agent for 3 h at room temperature, followed by incubation with a 1 mg/mL *Ld*NH solution for 16 h at 4 °C. Unbound enzymes in the supernatant were removed and stored for quantification. Finally, the system was incubated with a glycine solution (1.0 mol/L, pH 8.0) for 30 min at 4 °C. Then, the *Ld*NH-MNP was stored in PBS at 4 °C until use.^12^

Immobilized enzyme quantification was performed by comparing the concentrations of the initial *Ld*NH solution and the supernatant using Lowry’s method, with measurements obtained in a JASCO spectrophotometer model V-730BIO. The difference in concentration between these solutions was used to determine the immobilization yield.^46^

### 4.6 *Ld*NH-MNPs Stability and Kinetic Assays

The catalytic activity of *Ld*NH in stability and kinetic assays was conducted in microtubes under 10 s of manual agitation. The enzymatic reaction was interrupted by applying an external magnetic field for 1 min, during which *Ld*NH-MNPs were retained on the microtube wall, facilitating the collection of the supernatant (reaction medium). The collected supernatant was analyzed by HPLC-DAD to determine enzymatic activity based on Hypo production.

The storage stability study was carried out using the following conditions: 750 μmol/L of Ino in PBS and 8 μg/mL of *Ld*NH-MNP in PBS. Enzymatic activity was assessed as described above and monitored weekly for 2 months (8 weeks), followed by monthly assessments for up to 5 months (20 weeks). All samples were prepared in triplicate.

For the kinetic study, a 5 mmol/L Ino stock solution was used to prepare a series of dilutions covering the concentration range of 10–4000 μmol/L. The enzymatic activity assay was conducted under the same conditions as described above. The quantification of Hypo production was used and the data was adjusted to the Michaelis–Menten model to determine the *K*_Mapp_ constant.

### 4.7 Functional Assays with *Ld*NH-MNP

#### 4.7.1 *Ld*NH Inhibition Assays Using B. laevifolia Extracts

An initial screening assay was conducted with B. laevifolia flower (Bl-EEF) and leaf (Bl-EEL) extracts at 200 μg/mL, with Ino at 500 μmol/L and *Ld*NH-MNP at 0.8 μg/mL. Blank samples, representing 100% enzyme activity, were prepared by replacing the extract solution with the respective solvent. The *Ld*NH inhibition percentage was calculated using eq 1

1 $$inhibition\;\%=100-\left(\frac{\left[{Hypo}_{sample}\right]}{\left[{Hypo}_{blank}\right]}\times 100\right)$$

The IC_50_ values for Bl-EEL and Bl-EEF were determined by constructing a dose–response curve, varying the extract concentration from 0.5 to 200 μg/mL. Each experiment was performed in triplicate using Ino at 500 μmol/L and *Ld*NH-MNP at 0.8 μg/mL. The inhibition percentage for each concentration was calculated using eq 1, and nonlinear regression was performed using GraphPad Prism 8 software.

#### 4.7.2 *Ld*NH Inhibition Profiling for B. laevifolia Leaf Extract

Initially, an HPLC-DAD method was developed to obtain the chemical profile of the leaf extract of B. laevifolia using an HPLC-DAD system from Shimadzu (Kyoto, Japan), which included an LC 20AD XR pump, an SIL 10 AD VP autoinjector, and an SPD-M20A diode array detector. An INERTSUSTAIN C18 column (250 × 4.6 mm, 5 μm) was used with a gradient elution employing acetonitrile (solvent A) and ultrapure H_2_O (solvent B), both acidified with 0.1% formic acid at 0.8 mL/min, and an injection volume of 20 μL. The extract was analyzed using the following multistep gradient: 0–1 min: 10% A; 1–4 min: 10–15% A; 4–60 min: 15–30% A; 60–80 min: 30–45% A; 80–82 min: 45–100% A; 82–93 min: 100% A; 93–95 min: 100–10% A; 95–105 min: 10%.

Once the chemical profile was established, the selected extract was subjected to microfractionation. A 10.0 mg/mL solution of Bl-EEL extract was prepared in a MeOH/H_2_O (1:1, v/v) mixture. The sample was fractionated between 8 and 35 min of analysis, collecting the eluate every 15 s. After collection, all fractions were dried using a Cole–Parmer vacuum oven (model StableTemp, Vernon Hills, USA) set to 42 °C and 90 kPa vacuum.

Each dried microfraction was subjected to an inhibition assay using Ino at 500 μmol/L and *Ld*NH-MNP at 0.8 μg/mL. Control samples were prepared by microfractioning only the solvent used in the preparation of the extract solution, following the same chromatographic method and procedure. The percent inhibition for each microfraction was calculated using eq 1. *Ld*NH inhibition profiling was represented by plotting the percent inhibition against the retention time of each microfraction, resulting in a biochromatogram.

### 4.8 Nonfunctional Assay (AS-MS)

An affinity selection-mass spectrometry (AS-MS) assay was performed to identify ligands in the B. laevifolia leaf extract (Bl-EEL). To initiate the process, 2.5 mg of *Ld*NH-MNPs were incubated with 500 μL of a 4.0 mg/mL Bl-EEL solution prepared in 5 mmol/L ammonium acetate buffer (pH 7.4) for 15 min at room temperature with gentle agitation. Following magnetic extraction, the supernatant (S0) was removed. The *Ld*NH-MNPs were subsequently washed twice with 500 μL of ammonium acetate buffer (5 mmol/L, pH 7.4), yielding supernatants S1 and S2. Finally, the *Ld*NH-MNPs were incubated with 500 μL of MeOH for 25 min to elute the ligands. After magnetic extraction, the final supernatant (S3) was collected.

In parallel, the same procedure was carried out using inactive *Ld*NH-MNP, used as a control assay. For enzyme inactivation, *Ld*NH-MNPs were incubated with MeOH for 2 h at room temperature prior to AS-MS assay.

The Bl-EEL extract and the supernatants S0 and S3 from active and control assays were analyzed using the developed HPLC-DAD method described in Section 4.7.2. This method was transferred to a UHPLC-HRMS system (Maxis Impact, Bruker Daltonics, Billerica, USA). A flow split was used to achieve a flow rate of 0.3 mL/min into the mass spectrometer. The MS system included a quadrupole time-of-flight (QqTOF) analyzer operating in DDA/AutoMS acquisition mode with a scan range of 100–1000 *m*/*z* in positive ESI mode. Other MS parameters used were capillary voltage of 3500 V, nebulizer at 4 bar, dry gas at 8 L/min, dry temperature at 200 °C, and collision cell energy operated at 6 eV.

All data gathered were analyzed using Compass Data Analysis software and processed using MZmine (version 3 v3.9). The peak areas obtained from the total ion chromatogram (TIC) were used to determine the affinity ratio (AR). The AR of each ligand was calculated according to eq 2. Values of AR ≥ 1.2 were used as a threshold for ligand annotation.

2 $$AR=\frac{S_{3}\;{ligand\;area}_{LdNH\;active}}{S_{3}\;{ligand\;area}_{LdNH\;inactive}}$$

Four selective ligands recognized in the AS-MS assay were evaluated as *Ld*NH inhibitors. The flavonoids rutin, isoquercetin, orientin, and astragalin were initially screened at 100 μmol/L. Then, IC_50_ values for the most promising inhibitors were assessed by varying the inhibitor concentration from 5 to 350 μmol/L. Flavonoids are known to exhibit promiscuous inhibition due to their tendency to form aggregates. These aggregates can nonspecifically inhibit enzymes, leading to inaccurate results and overestimation of inhibitory potency. To minimize this effect and ensure a more reliable evaluation of inhibitory activity, 0.1% (v/v) Triton X-100 was added to the reaction medium to disrupt aggregate formation. The dose–response curve was obtained by nonlinear regression using GraphPad Prism 8 software.

## Supplementary Material



## Notes

Additional experimental data, including support characterization, chromatographic method validation for inosine and hypoxanthine separation, kinetic study, and structural annotations of the recognized ligands as well as components of the BL-EEL extract (PDF)

## References

[1] Alvar J., Yactayo S., Bern C. (2006). Leishmaniasis and poverty. Trends Parasitol..

[2] Kumar P., Kumar P., Singh N., Khajuria S., Patel R., Rajana V. K., Mandal D., Velayutham R. (2022). Limitations
of current chemotherapy and future of nanoformulation-based AmB delivery
for visceral leishmaniasis—An updated review. Front. Bioeng. Biotechnol..

[3] Figueroa-Villar J. D., Sales E. M. (2017). The importance of
nucleoside hydrolase enzyme (NH)
in studies to treatment of Leishmania: A review. Chem.-Biol. Interact..

[4] Newman D. J., Cragg G. M. (2020). Natural Products
as Sources of New Drugs over the Nearly
Four Decades from 01/1981 to 09/2019. J. Nat.
Prod..

[5] Muchiri R. N., Van Breemen R. B. (2021). Drug discovery
from natural products using affinity
selection-mass spectrometry. Drug Discovery
Today: Technol..

[6] Cragg G. M., Newman D. J. (2005). Biodiversity: A
continuing source of novel drug leads. Pure
Appl. Chem..

[7] Nirma C., Rangel G. T., Alves M. A., Casanova L. M., Moreira M. M., Rodrigues L. M., Hamerski L., Tinoco L. W. (2019). New *Leishmania
donovani* nucleoside hydrolase inhibitors from Brazilian flora. RSC Adv..

[8] Casanova L. M., Rodrigues L. M., De Aguiar P. F., Tinoco L. W. (2020). An NMR-Based Chemometric
Strategy to Identify *Leishmania donovani* Nucleoside
Hydrolase Inhibitors from the Brazilian Tree *Ormosia arborea*. J. Nat. Prod..

[9] Tabrez S., Rahman F., Ali R., Alouffi A. S., Alshehri B. M., Alshammari F. A., Alaidarous M. A., Banawas S., Bin Dukhyil A. A., Rub A. (2021). Assessment of the Antileishmanial
Potential of *Cassia fistula* Leaf Extract. ACS Omega.

[10] Muchiri R. N., Van Breemen R. B. (2021). Affinity
selection–mass spectrometry for the
discovery of pharmacologically active compounds from combinatorial
libraries and natural products. J. Mass Spectrom..

[11] Miranda
de Souza Duarte-Filho L. A., Ortega de Oliveira P. C., Yanaguibashi Leal C. E., de Moraes M. C., Picot L. (2023). Ligand fishing as a
tool to screen natural products with anticancer potential. J. Sep. Sci..

[12] de
Faria R. A., Oliveira P. C. O., de Carvalho M. D. P., Peixoto B. S., Severino V. G. P., Tinoco L. W., Rodrigues S. V., de Moraes M. C. (2022). High-resolution inhibition profiling and ligand fishing
for screening of nucleoside hydrolase ligands in Moringa oleifera
Lamarck. J. Pharm. Biomed. Anal..

[13] Domingo-Fernández D., Gadiya Y., Preto A. J., Krettler C. A., Mubeen S., Allen A., Healey D., Colluru V. (2024). Natural Products Have
Increased Rates of Clinical Trial Success throughout the Drug Development
Process. J. Nat. Prod..

[14] Shokrollahi H. (2017). A review of
the magnetic properties, synthesis methods and applications of maghemite. J. Magn. Magn. Mater..

[15] Ximenes I. A. T., Albino M., Sangregorio C., Cass Q. B., de Moraes M. C. (2022). On-flow
magnetic particle activity assay for the screening of human purine
nucleoside phosphorylase inhibitors. J. Chromatogr.
A.

[16] Gkantzou E., Chatzikonstantinou A. V., Fotiadou R., Giannakopoulou A., Patila M., Stamatis H. (2021). Trends in
the development of innovative
nanobiocatalysts and their application in biocatalytic transformations. Biotechnol. Adv..

[17] Ximenes I. A. T., de Oliveira P. C. O., Wegermann C. A., de Moraes M. C. (2021). Magnetic particles for enzyme immobilization:
A versatile
support for ligand screening. J. Pharm. Biomed.
Anal..

[18] Copeland, R. A. Enzymes: A Practical Introduction to Structure, Mechanism, and Data Analysis, 3rd ed.; Wiley: Hoboken, NJ, 2023.

[19] Spasojević M., Prodanović O., Pantić N., Popović N., Balaž A. M., Prodanović R. (2020). The Enzyme Immobilization: Carriers
and Immobilization methods. J. Eng. Process.
Manage..

[20] Zdarta J., Meyer A., Jesionowski T., Pinelo M. (2018). A General Overview
of Support Materials for Enzyme Immobilization: Characteristics, Properties,
Practical Utility. Catalysts.

[21] Kim K. S., Lee Y., Lee J. H., Lee S. S., Chung J. M., Jung H. S. (2024). Optimizing
protein crosslinking control: Synergistic quenching effects of glycine,
histidine, and lysine on glutaraldehyde reactions. Biochem. Biophys. Res. Commun..

[22] Rennó M. N., França T. C. C., Nico D., Palatnik-de-Sousa C.
B., Tinoco L. W., Figueroa-Villar J. D. (2012). Kinetics and docking studies of two
potential new inhibitors of the nucleoside hydrolase from Leishmania
donovani. Eur. J. Med. Chem..

[23] Copeland, R. A. Evaluation of Enzyme Inhibitors in Drug Discovery: A Guide for Medicinal Chemists and Pharmacologists, 1st ed.; Wiley, 2013. 16350889

[24] Oliveira D.
M., Silva T. F. R., Martins M. M., de Morais S. A. L., Chang R., de Aquino F. J. T., da Silva C. V., Teixeira T. L., Martins C. H. G., Moraes T. S., Cunha L. C. S., Pivatto M., de Oliveira A. (2018). Antifungal
and cytotoxicity activities of Banisteriopsis
argyrophylla leaves. J. Pharm. Pharmacol..

[25] Samoylenko V., Rahman Md.M., Tekwani B. L., Tripathi L. M., Wang Y.-H., Khan S. I., Khan I. A., Miller L. S., Joshi V. C., Muhammad I. (2010). Banisteriopsis caapi, a unique combination of MAO inhibitory
and antioxidative constituents for the activities relevant to neurodegenerative
disorders and Parkinson’s disease. J.
Ethnopharmacol..

[26] Wang Y.-H., Samoylenko V., Tekwani B. L., Khan I. A., Miller L. S., Chaurasiya N. D., Rahman Md.M., Tripathi L. M., Khan S. I., Joshi V. C., Wigger F. T., Muhammad I. (2010). Composition, standardization
and chemical profiling of Banisteriopsis caapi, a plant for the treatment
of neurodegenerative disorders relevant to Parkinson’s disease. J. Ethnopharmacol..

[27] de
Moraes M. C., Cardoso C. L., Cass Q. B. (2019). Solid-Supported
Proteins in the Liquid Chromatography Domain to Probe Ligand-Target
Interactions. Front. Chem..

[28] Schmid R., Heuckeroth S., Korf A., Smirnov A., Myers O., Dyrlund T. S., Bushuiev R., Murray K. J., Hoffmann N., Lu M., Sarvepalli A., Zhang Z., Fleischauer M., Dührkop K., Wesner M., Hoogstra S. J., Rudt E., Mokshyna O., Brungs C., Ponomarov K., Mutabdžija L., Damiani T., Pudney C. J., Earll M., Helmer P. O., Fallon T. R., Schulze T., Rivas-Ubach A., Bilbao A., Richter H., Nothias L.-F., Wang M., Orešič M., Weng J.-K., Böcker S., Jeibmann A., Hayen H., Karst U., Dorrestein P. C., Petras D., Du X., Pluskal T. (2023). Integrative analysis
of multimodal mass spectrometry data in MZmine 3. Nat. Biotechnol..

[29] Wang M., Carver J. J., Phelan V. V., Sanchez L. M., Garg N., Peng Y., Nguyen D. D., Watrous J., Kapono C. A., Luzzatto-Knaan T., Porto C., Bouslimani A., Melnik A. V., Meehan M. J., Liu W.-T., Crüsemann M., Boudreau P. D., Esquenazi E., Sandoval-Calderón M., Kersten R. D., Pace L. A., Quinn R. A., Duncan K. R., Hsu C.-C., Floros D. J., Gavilan R. G., Kleigrewe K., Northen T., Dutton R. J., Parrot D., Carlson E. E., Aigle B., Michelsen C. F., Jelsbak L., Sohlenkamp C., Pevzner P., Edlund A., McLean J., Piel J., Murphy B. T., Gerwick L., Liaw C.-C., Yang Y.-L., Humpf H.-U., Maansson M., Keyzers R. A., Sims A. C., Johnson A. R., Sidebottom A. M., Sedio B. E., Klitgaard A., Larson C. B., Boya
P C. A., Torres-Mendoza D., Gonzalez D. J., Silva D. B., Marques L. M., Demarque D. P., Pociute E., O’Neill E. C., Briand E., Helfrich E. J. N., Granatosky E. A., Glukhov E., Ryffel F., Houson H., Mohimani H., Kharbush J. J., Zeng Y., Vorholt J. A., Kurita K. L., Charusanti P., McPhail K. L., Nielsen K. F., Vuong L., Elfeki M., Traxler M. F., Engene N., Koyama N., Vining O. B., Baric R., Silva R. R., Mascuch S. J., Tomasi S., Jenkins S., Macherla V., Hoffman T., Agarwal V., Williams P. G., Dai J., Neupane R., Gurr J., Rodríguez A. M.
C., Lamsa A., Zhang C., Dorrestein K., Duggan B. M., Almaliti J., Allard P.-M., Phapale P., Nothias L.-F., Alexandrov T., Litaudon M., Wolfender J.-L., Kyle J. E., Metz T. O., Peryea T., Nguyen D.-T., VanLeer D., Shinn P., Jadhav A., Müller R., Waters K. M., Shi W., Liu X., Zhang L., Knight R., Jensen P. R., Palsson B. . Ø., Pogliano K., Linington R. G., Gutiérrez M., Lopes N. P., Gerwick W. H., Moore B. S., Dorrestein P. C., Bandeira N. (2016). Sharing and community curation of mass spectrometry
data with Global Natural Products Social Molecular Networking. Nat. Biotechnol..

[30] Shen N., Wang T., Gan Q., Liu S., Wang L., Jin B. (2022). Plant flavonoids: Classification,
distribution, biosynthesis, and
antioxidant activity. Food Chem..

[31] Kumar S., Pandey A. K. (2013). Chemistry and Biological
Activities of Flavonoids:
An Overview. Sci. World J..

[32] Li C., Dai T., Chen J., Li X., Li T., Liu C., McClements D. J. (2021). Protein-polyphenol functional ingredients: The foaming
properties of lactoferrin are enhanced by forming complexes with procyanidin. Food Chem..

[33] Rue E. A., Rush M. D., Van Breemen R. B. (2018). Procyanidins:
a comprehensive review
encompassing structure elucidation via mass spectrometry. Phytochem. Rev..

[34] Wei L., Ma R., Fu Y. (2022). Differences
in Chemical Constituents between Dalbergia
oliveri Heartwood and Sapwood and Their Effect on Wood Color. Molecules.

[35] Pereira C. A. M., Yariwake J. H., McCullagh M. (2005). Distinction
of theC-glycosylflavone
isomer pairs orientin/isoorientin and vitexin/isovitexin using HPLC-MS
exact mass measurement and in-source CID. Phytochem.
Anal..

[36] Xiao X., Xu L., Hu H., Yang Y., Zhang X., Peng Y., Xiao P. (2017). DPPH Radical
Scavenging and Postprandial Hyperglycemia Inhibition
Activities and Flavonoid Composition Analysis of Hawk Tea by UPLC-DAD
and UPLC-Q/TOF MSE. Molecules.

[37] Azizah M., Pripdeevech P., Thongkongkaew T., Mahidol C., Ruchirawat S., Kittakoop P. (2020). UHPLC-ESI-QTOF-MS/MS-Based
Molecular Networking Guided
Isolation and Dereplication of Antibacterial and Antifungal Constituents
of Ventilago denticulata. Antibiotics.

[38] Sapkota B. K., Khadayat K., Aryal B., Bashyal J., Jaisi S., Parajuli N. (2022). LC-HRMS-Based Profiling: Antibacterial
and Lipase Inhibitory
Activities of Some Medicinal Plants for the Remedy of Obesity. Sci. Pharm..

[39] Ye L.-H., He X.-X., Yan M.-Z., Chang Q. (2014). Identification of in
vivo components in rats after oral administration of lotus leaf flavonoids
using ultrafast liquid chromatography with tandem mass spectrometry. Anal. Methods.

[40] Zavilopulo A. N., Shpenik O. B., Mylymko A. N., Shpenik V. Yu. (2019). Mass spectrometry
of d-ribose molecules. Int. J. Mass Spectrom..

[41] Da
Silva J. M. G., De Almeida R. F., Zeraik M. L. (2024). Comparative Metabolite
Profiling of Three Savannic Species of Banisteriopsis (Malpighiaceae)
via UPLC-MS/MS and Chemometric Tools. Chem.
Biodiversity.

[42] Alexandre G. P., Simão J. L. S., Tavares M. O. A., Zuffo I. M. S., Prado S. V., Paiva J. A. D., Mustapha A. N., Oliveira A. E. D., Kato L., Severino V. G. P. (2022). Dereplication by HPLC-ESI-MS and antioxidant activity
of phenolic compounds from Banisteriopsis laevifolia (Malpighiaceae). An. Acad. Bras. Cienc.

[43] Liu X. L., Fan H. M., Yi J. B., Yang Y., Choo E. S. G., Xue J. M., Fan D. D., Ding J. (2012). Optimization of surface
coating on Fe3O4 nanoparticles for high performance magnetic hyperthermia
agents. J. Mater. Chem..

[44] Borri C., Albino M., Innocenti C., Pineider F., Cavigli L., Centi S., Sangregorio C., Ratto F., Pini R. (2020). A bionic shuttle
carrying multi-modular particles and holding tumor-tropic features. Mater. Sci. Eng., C.

[45] Basini M., Guerrini A., Cobianchi M., Orsini F., Bettega D., Avolio M., Innocenti C., Sangregorio C., Lascialfari A., Arosio P. (2019). Tailoring the magnetic
core of organic-coated
iron oxides nanoparticles to influence their contrast efficiency for
Magnetic Resonance Imaging. J. Alloys Compd..

[46] Lowry O. H., Rosebrough N. J., Farr A. L., Randall R. J. (1951). Protein measurement
with the Folin phenol reagent. J. Biol. Chem..

