# Supplementary material for: Identification of Novel Leishmania donovani Nucleoside Hydrolase Inhibitors from Banisteriopsis laevifolia Using Affinity Selection-Mass Spectrometry
Source: ACS Omega. 2025 May 23;10(22):23249–59. doi: 10.1021/acsomega.5c01613 (PMC12163827; doi:10.1021/acsomega.5c01613)
Supplement: Supplementary file 1 [file ao5c01613_si_001.pdf]

Identification of novel *Leishmania donovani* nucleoside hydrolase inhibitors from *Banisteriopsis laevifolia* using affinity selection-mass spectrometry

Pamella C. O. de Oliveira[a], Pedro R. C. Medeiros[a], Bruno C. B. Marques[b], Jorge L. S. Simão[c], Martin Albino[d,e], Vanessa G. P. Severino[c], Claudio Sangregorio[d,e], Luzineide W. Tinoco[b], Marcela C. de Moraes[a,*]

[a]BioCrom, Organic Chemistry Department, Chemistry Institute, Fluminense Federal University, 24020-141, Niterói/RJ, Brazil

[b]Laboratory for Analysis and Development of Enzyme Inhibitors, Natural Products Research Institute, Federal University of Rio de Janeiro, 21941-902, Rio de Janeiro/RJ, Brazil

[c]Institute of Chemistry, Federal University of Goiás, Campus Samambaia, Esperança avenue, 74690-900 Goiânia, Brazil

[d]ICCOM-CNR, 50019 Sesto Fiorentino (FI), Italy

[e]Department of Chemistry 'Ugo Schiff', University of Florence and INSTM, 50019 Sesto Fiorentino (FI), Italy

*Corresponding author:
E-mail address: mcmoraes@id.uff.br (M.C. de Moraes).

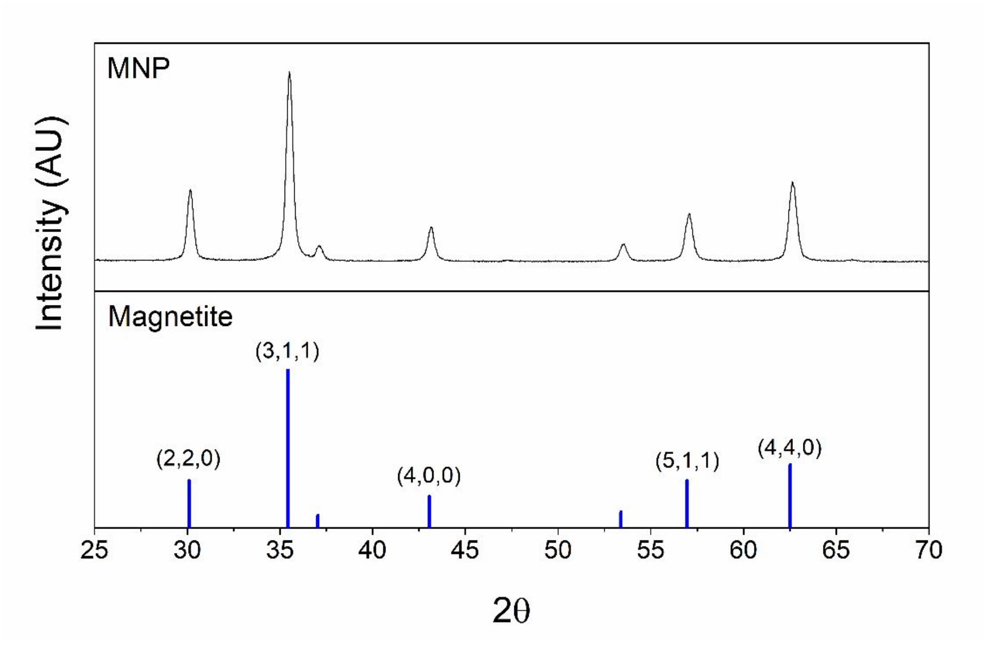

Figure S1: Diffractogram of synthesized magnetic nanoparticle (MNP) and X-ray diffraction patterns of magnetite (blue).

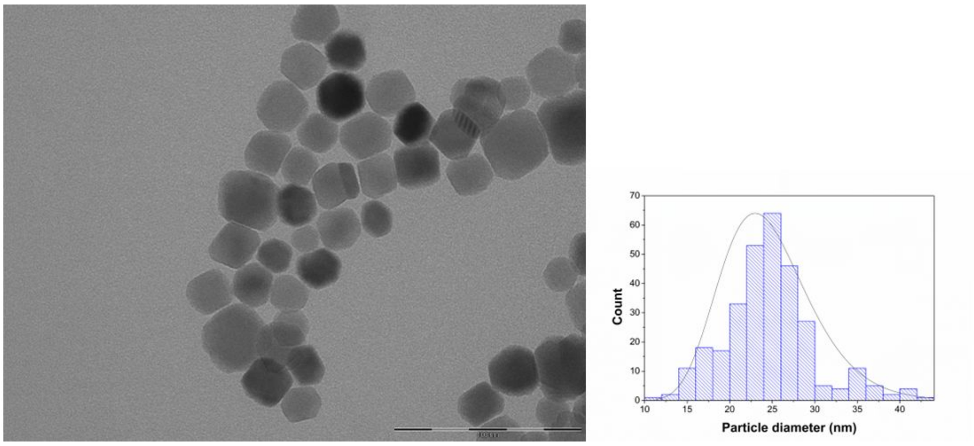

Figure S2: TEM micrograph of iron oxide MNPs, at a 100 nm scale, and their size distribution histogram, fitted to a lognormal function.

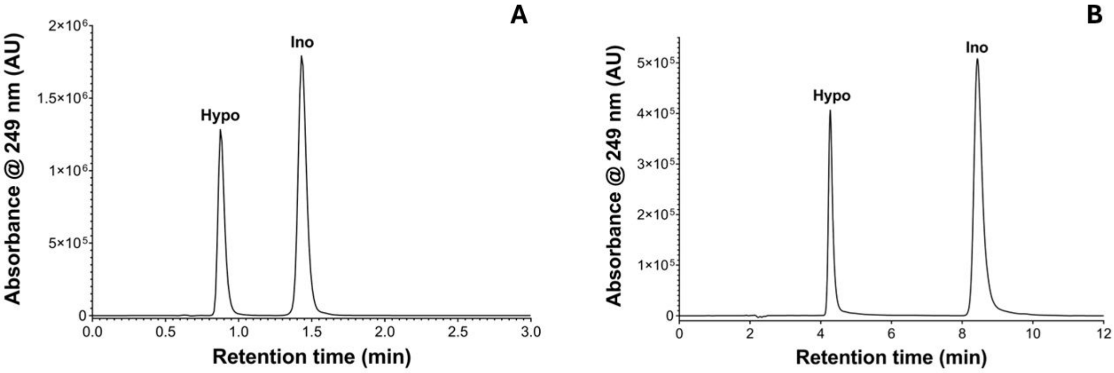

Figure S3: Chromatographic separation between the analytes Ino and Hypo. **A** – New chromatographic method optimized for 3 minutes; **B** - 12-minute chromatographic method.

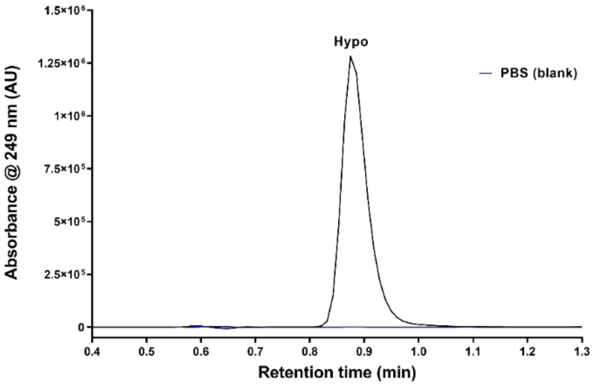

Figure S4: Selectivity of the 3-minute inosine/hypoxanthine separation method, through the comparison of analyte signal (Hypo) and blank, composed of PBS.

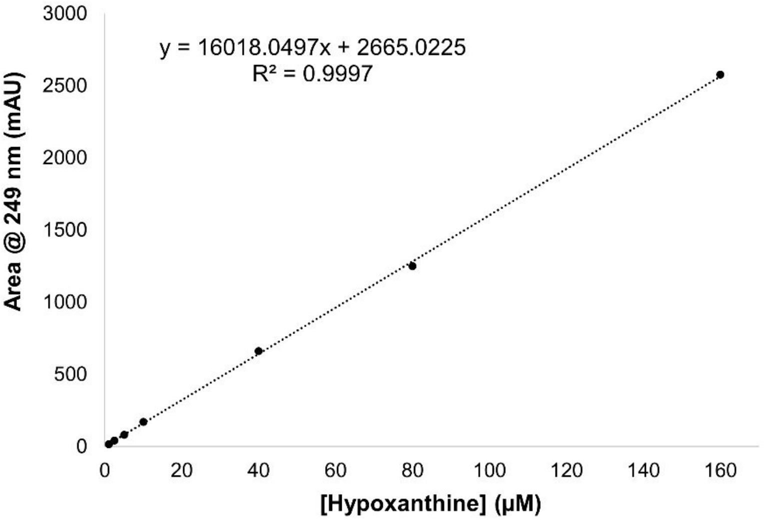

Figure S5: Analytical curve for hypoxanthine (Hypo)

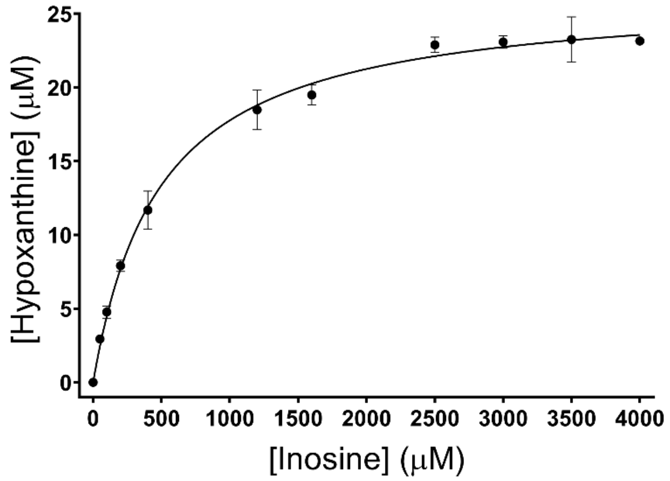

Figure S6: Kinetic study of immobilized *Ld*NH on magnetic nanoparticle (*Ld*NH-MNP).

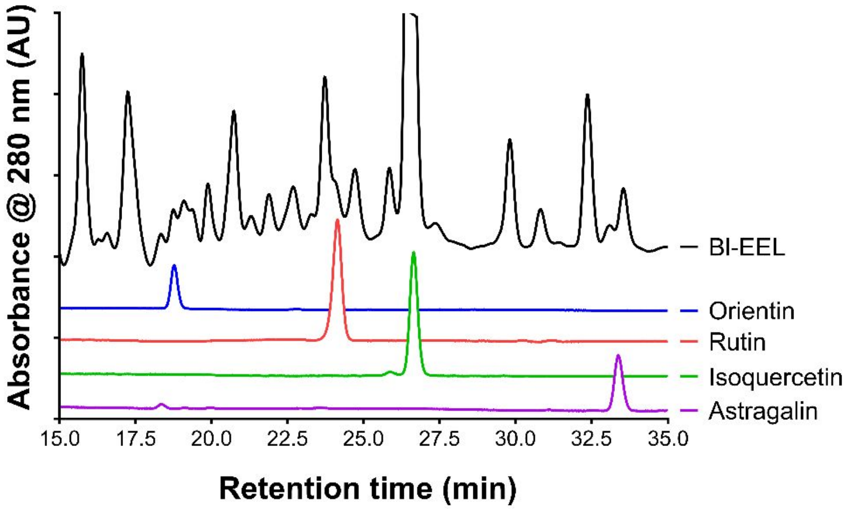

Figure S7: Superposition of chromatograms at 280 nm of BI-EEL (black), orientin (blue), rutin (red), isoquercetin (green), and astragalin (purple).

Table S1: Intra- and inter-day precision and accuracy of the optimized analytical method.

Control (μmol/L)	1st Day (n = 5)		2nd Day (n = 5)		3rd Day (n = 5)		Average (n = 15)	
	Accuracy (%)	CV (%)	Accuracy (%)	CV (%)	Accuracy (%)	CV (%)	Accuracy (%)	CV (%)
1.2	89.6	6.64	91.8	5.24	95.0	11.20	93.1	7.69
50	99.8	1.06	103.7	3.90	103.5	2.88	102.4	2.61
150	103.1	3.01	103.7	1.33	103.4	1.83	103.4	2.06

Table S2: LC-HRMS/MS data in positive mode and structure annotation for the *Ld*NH ligands identified through AS-MS assay.

	RT (min)	AR	Ligand	m/z	Molecular ion	Molecular Formula	Proposed structure	Fragments (m/z)
1	12.40	2.30	procyanidin B2	579.1506	M+H	$C_{30}H_{26}O_{12}$	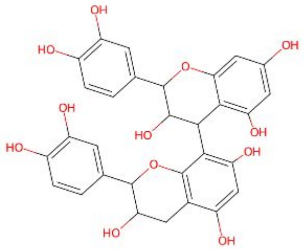	289.0694<br>127.0397<br>291.0856<br>579.1458
2	14.31	1.20	(-)-epicatechin	291.0863	M+H	$C_{15}H_{14}O_6$	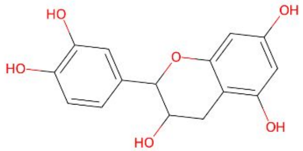	139.0387<br>291.0868<br>123.0438<br>165.0543
3	16.83	2.30	orientin	449.1078	M+H	$C_{21}H_{20}O_{11}$	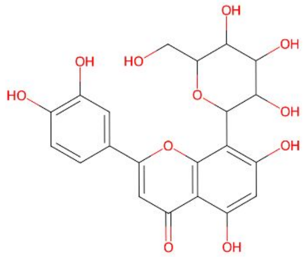	329.0657<br>413.0869<br>431.0975<br>449.1081

4	18.44	2.22	isoorientin	449.1079	M+H	$C_{21}H_{20}O_{11}$	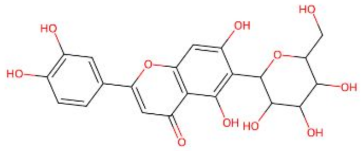	329.0667<br>299.0546<br>353.0650<br>413.0975
5	20.36	3.09	-	597.1457	-	-	-	303.0507<br>304.0532<br>465.1036<br>305.0549<br>115.0411
6	23.29	3.51	rutin	611.1614	M+H	$C_{27}H_{30}O_{16}$	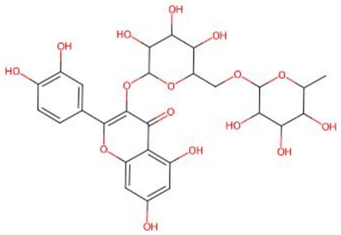	303.0499<br>304.0531<br>465.1028<br>129.0551
7	24.61	2.20	-	579.1501	-	-	-	409.0917<br>287.0549<br>289.0707<br>123.0450<br>275.0554
8	25.51	2.19	isoquercetin	465.1028	M+H	$C_{21}H_{20}O_{12}$	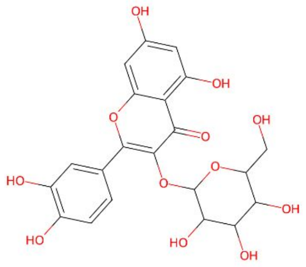	303.0495<br>304.0531<br>305.0544<br>301.0340

9	26.22	1.40	quercetin 3-*O*-glucuronide	479.0823	M+H	$C_{21}H_{18}O_{13}$	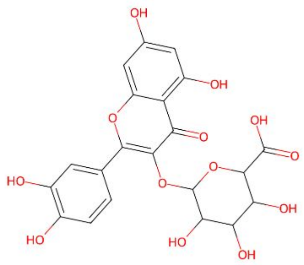	303.0497<br>479.0826<br>113.0231<br>85.0295
10	29.39	2.50	2-(3,4-dihydroxyphenyl)-5,7-dihydroxy-3-[(2S,3R,4S,5S)-4-hydroxy-5-(hydroxymethyl)-3-[(2S,3R,4S,5R)-3,4,5-trihydroxyoxan-2-yl]oxyoxolan-2-yl]oxychromen-4-one	567.1344	M+H	$C_{25}H_{26}O_{15}$	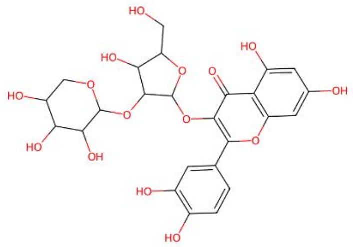	303.0506<br>304.0527<br>115.0402<br>97.03060<br>133.0504
11	33.52	4.70	astragalin	449.1071	M+H	$C_{21}H_{20}O_{11}$	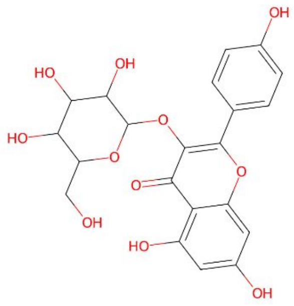	287.0553<br>288.0586<br>289.0614<br>451.1134<br>449.1083

Table S3: LC-HRMS/MS data in positive mode of *B. laevifolia* leaf crude extract (BL-EEL) annotated using GNPS.

	RT (min)	Name	*m/z*	Molecular ion	Molecular Formula	Fragments (*m/z*)
1	4.68	adenosine	268.1041	M+H	$C_{10}H_{13}N_5O_4$	136.0626<br>268.1044<br>269.1073<br>137.0647
2	10.73	chlorogenic acid	355.1024	M+H	$C_{16}H_{18}O_9$	163.0394<br>164.0432<br>145.0299<br>135.0436
3	11.51	catechin	291.0860	M+H	$C_{15}H_{14}O_6$	139.0397<br>291.0863<br>123.0449<br>165.0552
4	17.07	3-[(2S,3R,4S,5S,6R)-6-[[(2R,3R,4R,5R,6S)-3-[(2S,3R,4R)-3,4-dihydroxy-4-(hydroxymethyl)oxolan-2-yl]oxy-4,5-dihydroxy-6-methyloxan-2-yl]oxymethyl]-3,4,5-trihydroxyoxan-2-yl]oxy-2-(3,4-dihydroxyphenyl)-5,7-dihydroxychromen-4-one	743.2043	M+H	$C_{32}H_{38}O_{21}$	303.0498<br>304.0530<br>465.1038<br>129.0559
5	22.58	vitexin	433.1130	M+H	$C_{21}H_{20}O_{10}$	433.1131<br>313.0699<br>397.0932<br>434.1171<br>283.0606
6	23.12	3-genistein-8-*C*-glucoside	433.1132	M+H	$C_{21}H_{20}O_{10}$	313.0701<br>433.1139<br>283.0599<br>415.1026<br>337.0703
7	25.58	plantaginin	449.1082	M+H	$C_{21}H_{20}O_{11}$	287.0551<br>288.0600<br>449.1104

8	26.29	5,7-dihydroxy-2-(4-hydroxyphenyl)-3-[(2S,3R,4S,5S,6R)-3,4,5-trihydroxy-6-[[(2S,3R,4S,5S)-3,4,5-trihydroxyoxan-2-yl]oxymethyl]oxan-2-yl]oxychromen-4-one	581.1507	M+H	$C_{26}H_{28}O_{15}$	287.0551<br>288.0581<br>449.1085<br>289.0604<br>97.0313
9	27.37	procyanidin A2	577.1350	M+H	$C_{30}H_{24}O_{12}$	287.0548<br>425.0879<br>288.0566<br>437.0894
10	29.35	kaempferol-7-neohesperidoside	595.1663	M+H	$C_{27}H_{30}O_{15}$	287.0551<br>288.0575<br>449.1097<br>129.0547
11	30.30	narcissin	625.1773	M+H	$C_{28}H_{32}O_{16}$	317.0656<br>318.0678<br>303.0500<br>129.0565<br>479.1176
12	32.04	quercetin-3-*O*-pentoside	435.0926	M+H	$C_{20}H_{18}O_{11}$	303.0508<br>304.0531<br>133.0507<br>305.0557<br>115.0412
13	33.24	kaempferol 3-glucuronide	463.0878	M+H	$C_{21}H_{18}O_{12}$	287.0554<br>288.0581<br>113.0256<br>463.0882<br>159.0290
14	39.76	kaempferol-3-*O*-pentoside	419.0975	M+H	$C_{20}H_{18}O_{10}$	287.0553<br>288.0583<br>115.0383<br>133.0494
15	56.59	kaempferol-3-*O*-(6-p-coumaroyl)-glucoside	595.1455	M+H	$C_{30}H_{26}O_{13}$	147.0447

						287.0545<br>309.0960<br>291.0865
